# (-)-Epigallocatechin-3-Gallate Protects against NO-Induced Ototoxicity through the Regulation of Caspase- 1, Caspase-3, and NF-κB Activation

**DOI:** 10.1371/journal.pone.0043967

**Published:** 2012-09-28

**Authors:** Su-Jin Kim, Jeong-Han Lee, Beom-Su Kim, Hong-Seob So, Raekil Park, Noh-Yil Myung, Jae-Young Um, Seung-Heon Hong

**Affiliations:** 1 Department of Cosmeceutical Science, Daegu Hanny University, Kyungsan, Gyeoungbuk, Republic of Korea; 2 Center for Metabolic Function Regulation, Wonkwang University, Iksan, Republic of Korea; 3 Wonkwang Bone Regeneration Research Institute, Wonkwang University, Iksan, Jeonbuk, Republic of Korea; 4 College of Oriental Medicine, Kyung Hee University, Seoul, Republic of Korea; National Institutes of Health, United States of America

## Abstract

Excessive nitric oxide (NO) production is toxic to the cochlea and induces hearing loss. However, the mechanism through which NO induces ototoxicity has not been completely understood. The aim of this study was to gain further insight into the mechanism mediating NO-induced toxicity in auditory HEI-OC1 cells and in *ex vivo* analysis. We also elucidated whether and how epigallocatechin-3-gallate (EGCG), the main component of green tea polyphenols, regulates NO-induced auditory cell damage. To investigate NO-mediated ototoxicity, *S*-nitroso-*N*-acetylpenicillamine (SNAP) was used as an NO donor. SNAP was cytotoxic, generating reactive oxygen species, releasing cytochrome *c*, and activating caspase-3 in auditory cells. NO-induced ototoxicity also mediated the nuclear factor (NF)-κB/caspase-1 pathway. Furthermore, SNAP destroyed the orderly arrangement of the 3 outer rows of hair cells in the basal, middle, and apical turns of the organ of Corti from the cochlea of Sprague–Dawley rats at postnatal day 2. However, EGCG counteracted this ototoxicity by suppressing the activation of caspase-3/NF-κB and preventing the destruction of hair cell arrays in the organ of Corti. These findings may lead to the development of a model for pharmacological mechanism of EGCG and potential therapies against ototoxicity.

## Introduction

Nitric oxide (NO) plays essential roles in the physiological functions of the inner ear, including regulation of neurotransmission and blood flow [1]. Recently, accumulating evidence has suggested that excessive NO production may cause hearing impairment [2], [3]. Noise-induced hearing loss can be caused by increased NO production in the inner ear, leading to auditory cell destruction [4]–[6]. Previous studies have suggested that treating animals with ascorbic acid, an agent that attenuates noise-induced hearing loss, reduces the concentration of NO [7]. These results indicate that excessive NO production may play an important role in pathological damage to the cochlea and elevated hearing thresholds. Although the correlation between hearing loss and NO production has been described *in vitro* and *in vivo*, the mechanism through which NO mediates ototoxicity has not been completely understood.

Apoptosis is a process involving genetically regulated programmed cell death that plays an essential role in the development and homeostasis of higher organisms [8]. Mitochondria, the central coordinators of apoptotic events, are involved in the intrinsic pathway of apoptosis [9]. Mitochondria induce apoptosis by increasing mitochondrial membrane permeability and producing reactive oxygen species (ROS) [10]. A key event in apoptotic signaling is the release of pro-apoptotic proteins, including cytochrome *c* (cyt *c*) and apoptosis-inducing factor (AIF), from the mitochondrial intermembrane space [11], [12]. Once released, these proteins promote apoptosis through the activation of both caspase-dependent and caspase-independent pathways [13].

Nuclear factor kappa B (NF-κB) has been implicated in the regulation of proliferation, survival, angiogenesis, apoptosis, and differentiation [14]–[16]. In the nucleus, NF-κB activates genes that regulate apoptosis and respond to inflammation and oxidative stress [17], [18]. Many studies have reported the role of NF-κB in hearing loss. Ototoxic stimulants, such as noise exposure and ototoxic drugs, can induce NF-κB activation [19], [20], resulting in insults to the cochlear lateral wall via the production of high levels of ROS [21]–[23]. Acoustic overstimulation also increases the expression of inflammatory factors through NF-κB activation in the inner ear [24].

Caspase-1, a member of the caspase family that contains large prodomains [25], is involved in apoptosis and inflammation [26]. Caspase-1 activation induces inflammation via the production of pro-inflammatory cytokines [27], and caspase-1 also plays an important role in cisplatin-induced apoptosis in cochlear hair cells and spiral ganglion neurons [28]. However, the relationship between NO and caspase-1 activation in auditory cells has not yet been described.

**Figure 1:**
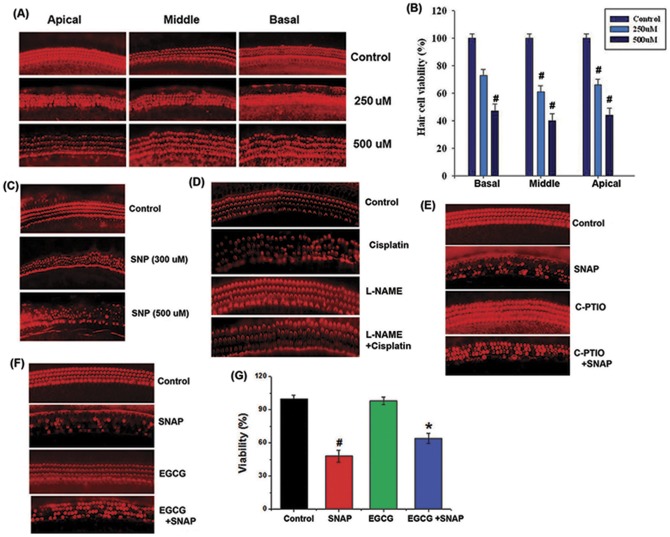
Effects of EGCG on organ of Corti explants. (A) Cochlear explants from the apical, middle, and basal turns of the rat cochlea were incubated with SNAP (250–500 μM) for 24 h. (B) Relative cell viability is shown. (C) Cochlear explants were incubated with SNP (300–500 μM) for 24 h. (D) Explants were treated with 200 μM L-NAME for 1 h and subsequently treated with 20 μM cisplatin. (E) Explants were treated with 10 μM C-PTIO for 1 h and subsequently treated with 500 μM SNAP. (F) Cochlea explants were treated with 50 μM EGCG and then 500 μM SNAP for 24 h. The explants were fixed with paraformaldehyde (4%), and TRITC-conjugated phalloidin (red), which binds to F-actin, was used to stain hair cells. (G) Relative cell viability is shown. All data represent the mean ± SEM of 3 independent experiments (*^#^P*<0.05 vs. control, *P<0.05 vs. SNAP alone).

Green tea, which contains a wide range of catechins, has a variety of modulatory effects on physiological functions, including antibacterial, radical scavenging, and antioxidant activities. Green tea also has a protective effect on the gastric mucosa and has been implicated in the prevention of atherosclerosis [29]. Epigallocatechin-3-gallate (EGCG), a major component of tea catechins, inhibits allergic reactions [30], [31] and penetrates the blood–brain barrier, making it a promising candidate for the treatment of neurodegenerative disorders. However, the otoprotective effects of EGCG in the context of NO damage remain unknown.

**Figure 2:**
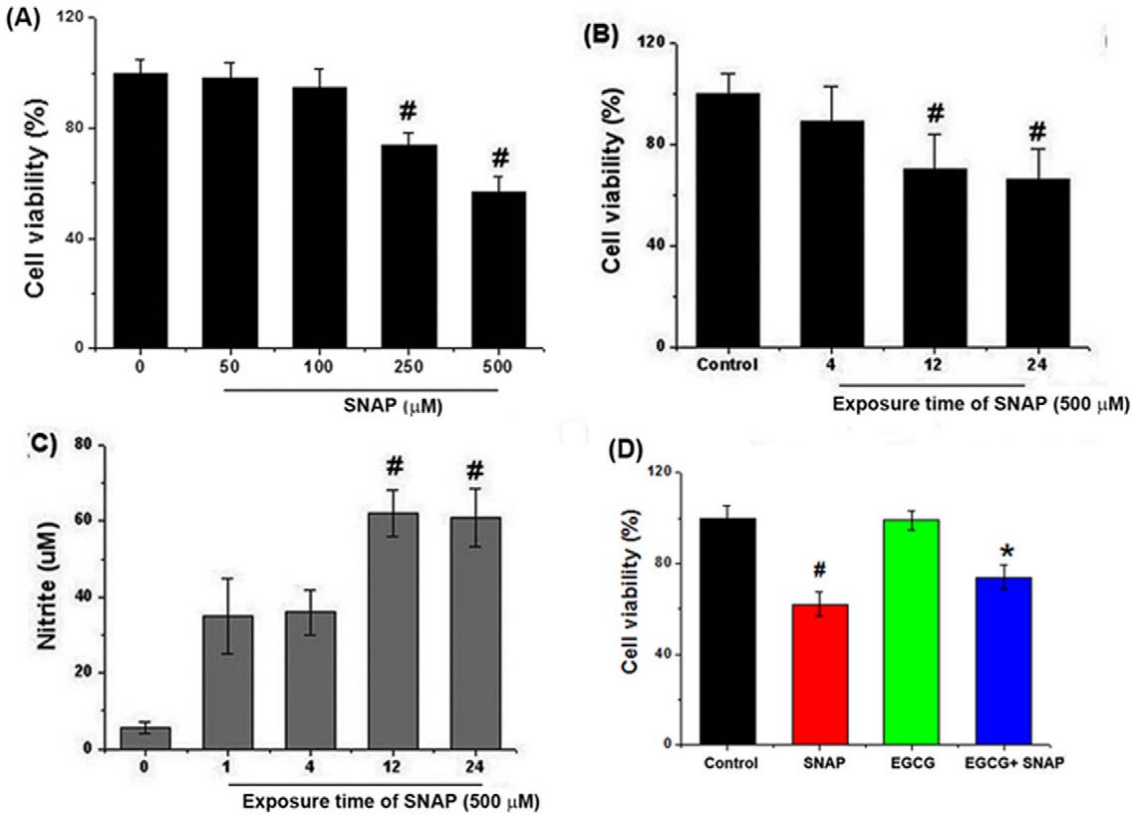
Effects of EGCG on NO-induced cell death in HEI-OC1 cells. (A, B) Cell viability evaluated by MTT colorimetric assay, as a function of SNAP concentration and exposure time. (C) Cells were treated with 500 μM SNAP for varying times, and NO levels were determined by the measurement of nitrite based on the Griess reaction. (D) Cells were pretreated with 50 μM EGCG and then 500 μM SNAP for 24 h. Cell viability was evaluated by MTT assay. All data represent the mean ± SEM of 3 independent experiments (*^#^P*<0.05 vs. control, *P<0.05 vs. SNAP alone).

The overall aim of this study was to gain further insight into the mechanism of NO-induced toxicity in auditory HEI-OC1 cells and in *ex vivo* analysis. We also examined whether and how EGCG regulates NO-induced auditory cell damage. The specific aims were as follows: (Ι) to examine the effects of NO on cell death, ROS generation, mitochondrial membrane potential (MMP) loss, cyt *c* release, caspase-3 activation, and NF-κB/caspase-1 activation in HEI-OC1 cells; (II) to investigate NO-induced damage to the arrangement of cochlear hair cells in the basal, middle, and apical turns of the organ of Corti from rats; and (III) to investigate the protective effects of EGCG against NO-induced ototoxicity both *in vitro* and *ex vivo*.

**Figure 3:**
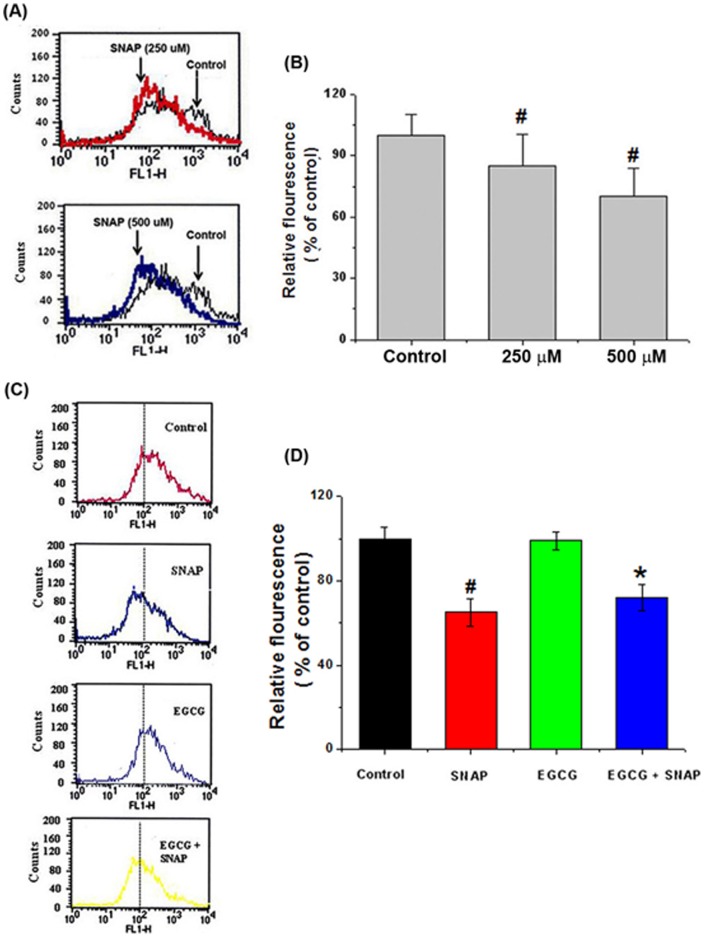
Effects of EGCG on NO-induced MMP loss in HEI-OC1 cells. (A) MMP levels were measured by flow cytometry using the fluorescent probe DiOC_6_. SNAP incubation (250–500 μM) resulted in a left shift of the cell distribution, indicating reduced MMP. (B) The mean fluorescence intensity of the traces is depicted in panel A. (C) Cells were pretreated with 50 μM EGCG, followed by treatment with 500 μM SNAP for 24 h. MMP levels were measured by flow cytometry. (D) The mean fluorescence intensity of the traces is depicted in panel C. All data represent the mean ± SEM of 3 independent experiments (*^#^P*<0.05 vs. control, *P<0.05 vs. SNAP alone).

## Materials and Methods

### Reagents

Fetal bovine serum (FBS) and high-glucose Dulbecco's modified Eagle medium (DMEM) were purchased from GIBCO BRL (Grand Island, NY). 3-[4,5-Dimethylthiazol-2-yl]-2,5-diphenyltetrazoliumbromide (MTT), dimethyl sulfoxide (DMSO), EGCG, and other reagents were obtained from Sigma (St. Louis, MO, USA). Cyt *c*, NF-κB (p65), IκB-α, and caspase-1 antibodies (Abs) were obtained from Santa Cruz Biotechnology (Santa Cruz, CA, USA). Voltage-dependent anion channel (VDAC) and caspase-3 Abs were purchased from Cell Signaling Technology, Inc. (Danvers, MA, USA). Caspase assay kits, anti-mouse interleukin (IL)-1β Abs, biotinylated IL-1β Abs, and recombinant mouse (rm) IL-1β Abs were purchased from R&D Systems Inc. (Minneapolis, MN, USA). *S*-Nitroso-*N*-acetylpenicillamine (SNAP) was purchased from Calbiochem (San Diego, CA, USA).

**Figure 4:**
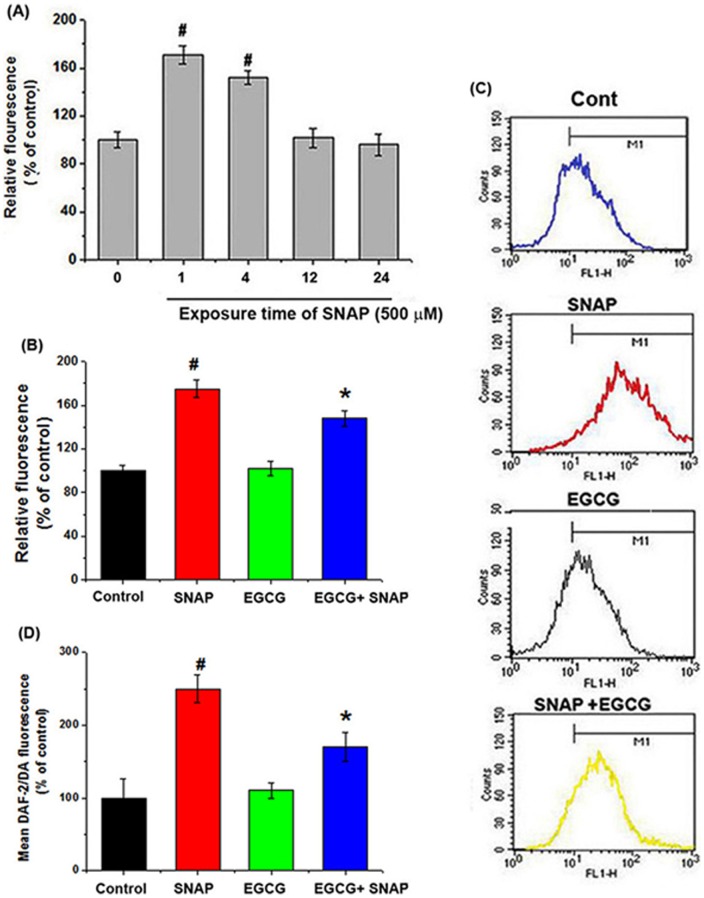
Effects of EGCG on NO-induced ROS production in HEI-OC1 cells. (A) ROS levels were measured using the fluorescent probe DCFH-DA and a spectrofluorometer. Cells were treated with 500 μM SNAP for varying times. (B) Cells were pretreated with 50 μM EGCG, followed by treatment with 500 μM SNAP for 1 h. The relative fluorescence levels were measured. (C) ROS production was measured by flow cytometry analysis. (D) The relative fluorescence levels of DAF-2/DA were measured. All data represent the mean ± SEM of 3 independent experiments (*^#^P*<0.05 vs. control, *P<0.05 vs. SNAP alone).

### Cell culture

The HEI-OC1 cell line was a gift from Dr. Federico Kalinec (House Ear Institute, CA, USA). HEI-OC1 cells express several molecular markers that are characteristic of sensory cells of the organ of Corti, including thyroid hormone, brain-derived neurotrophic factor, calbindin, calmodulin, Connexin 26, Math 1, Myosin 7a, organ of Corti protein 2, tyrosine kinase receptor B and C, platelet-derived growth factor receptor, and prestin. HEI-OC1 cells are also extremely sensitive to ototoxic drugs [32]. The cells were maintained in DMEM with 10% FBS at 33°C under 5% CO_2_ in air.

**Figure 5:**
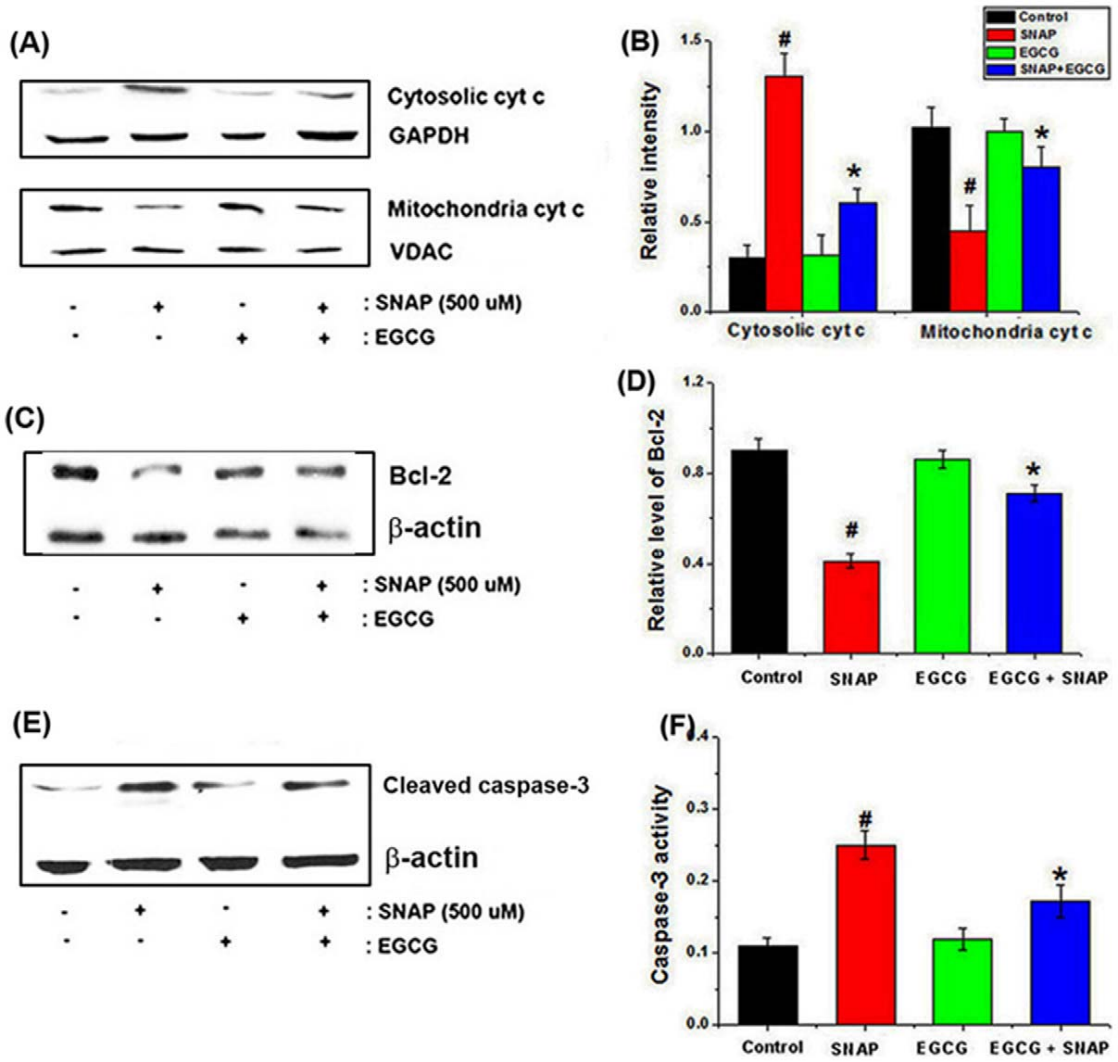
Effects of EGCG on NO-induced apoptosis-related genes in HEI-COΙ cells. (A) Cells were pretreated with 50 μM EGCG, followed by treatment with 500 μM SNAP for 24 h. After isolation of cytosolic and mitochondrial fractions, the protein extracts were assayed for cyt *c* by western blot analysis. GAPDH was used as an internal cytosolic marker control, and VDAC was used as a mitochondrial marker. (B) The relative levels of cytosolic and mitochondrial cyt *c* were quantified by densitometry. (C) Western blot analysis revealed a reduction in Bcl-2 protein after SNAP exposure. (D) Relative levels of Bcl-2 are shown. (E) The levels of cleaved caspase-3 after treatment with EGCG were assayed by western blot analysis. (F) The effect of EGCG on caspase-3 activation was determined using a colorimetric kit. All data represent the mean ± SEM of 3 independent experiments (*^#^P*<0.05 vs. control, *P<0.05 vs. SNAP alone).

### Ethics statement

All animal procedures and experiments were approved by the Animal Ethics Committee of Wonkwang University (approval number WKU10-038).

**Figure 6:**
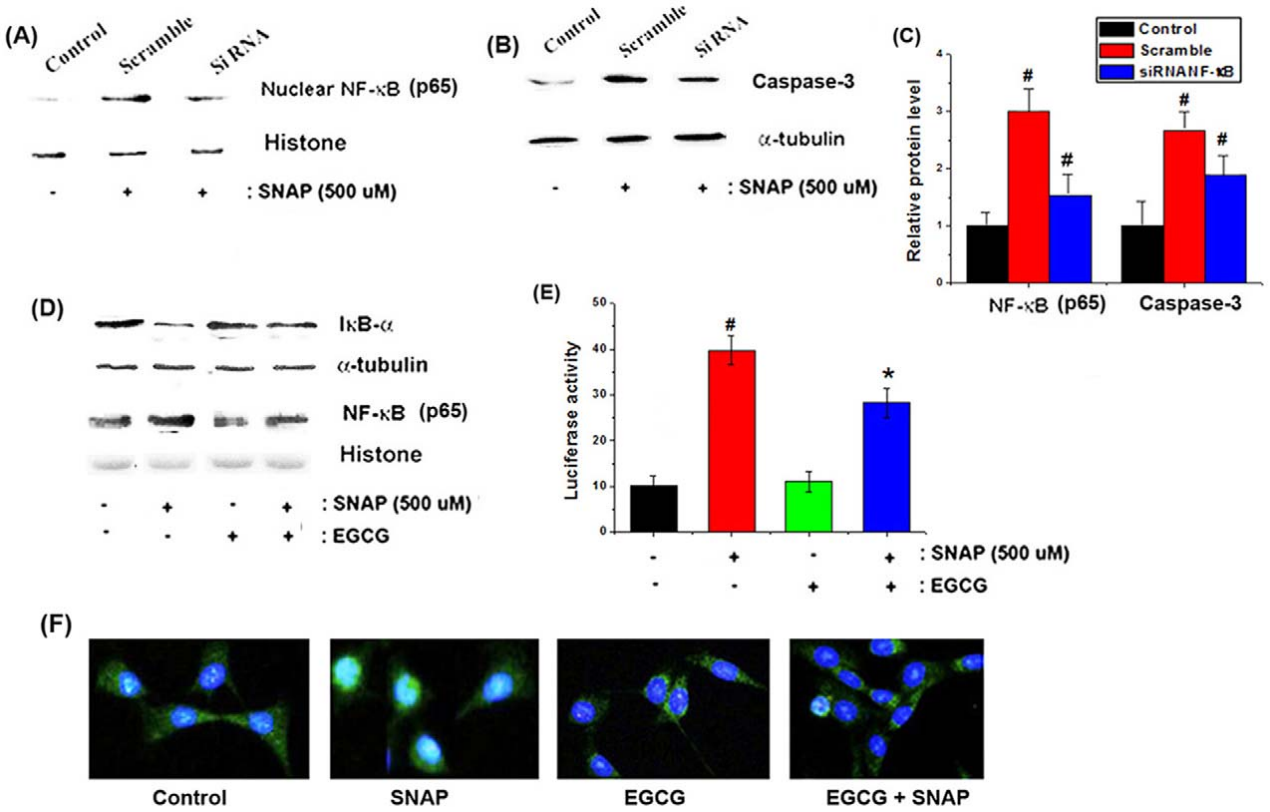
Effects of EGCG on NO-induced NF-κB activation in HEI-COΙ cells. (A) After transfection, cells were treated with 500 μM SNAP for 4 h. After isolation of the nuclear fraction, protein extracts were assayed for NF-κB activity by western blot analysis. (B) Protein extracts were assayed for caspase-3 by western blot analysis. (C) Relative levels of NF-κB and caspase-3 are shown. (D) Cells were pretreated with 50 μM EGCG, followed by treatment with 500 μM SNAP for 4 h. After isolation of cytosolic and nuclear fractions, protein extracts were assayed for IκB-α and NF-κB by western blot analysis. (E) Cells were transfected with an NF-κB-dependent reporter gene for 48 h, and transfected cells were treated with SNAP for 4 h. EGCG (50 μM) was added 2 h prior to SNAP treatment. Cells were harvested, and luciferase activity was measured as described in the Materials and Methods. (F) Cells were fixed and stained with NF-κB (green) and DAPI (blue). Cells were then analyzed using an Olympus microscope (magnification, 100×). All data represent the mean ± SEM of 3 independent experiments (*^#^P*<0.05 vs. control, *P<0.05 vs. SNAP alone).

**Figure 7:**
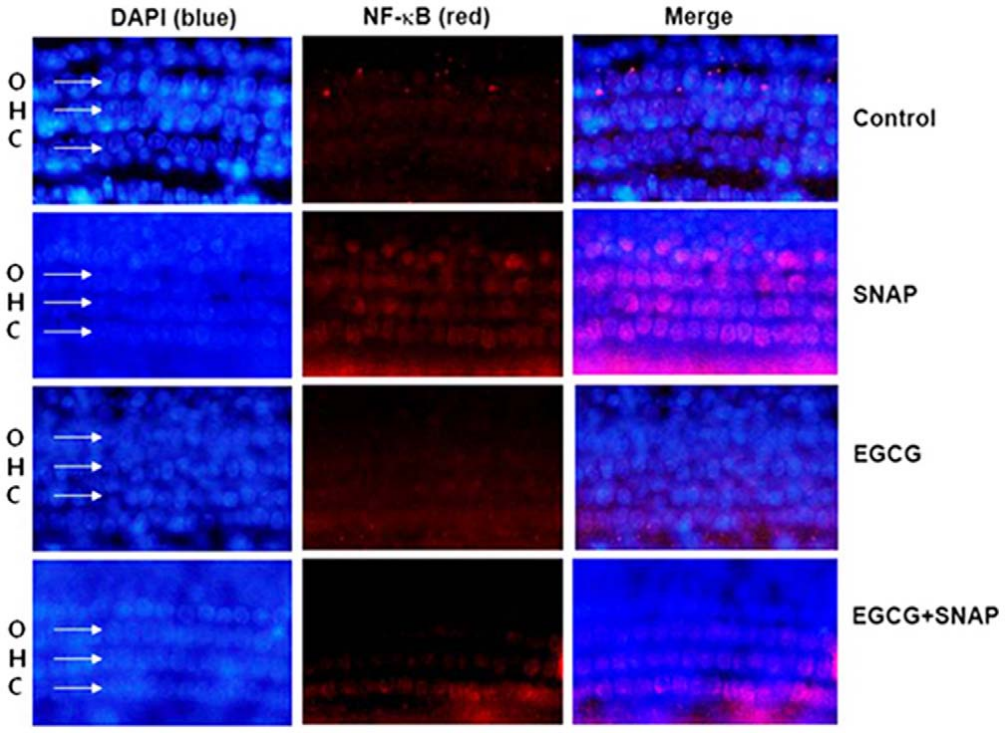
Effects of EGCG on NF-κB activation in the organ of Corti. Organ of Corti explants isolated from rats were pretreated with 50 μM EGCG for 2 h, followed by treatment with 500 μM SNAP for 4 h. Explants were fixed, stained with TRITC-conjugated phalloidin (NF-κB, red) and DAPI (nuclear, blue), and examined under an Olympus microscope (magnification, 100×).

### Organ of Corti explant cultures

Organ culturing procedures were similar to those described previously [33]. Sprague–Dawley rats were killed on postnatal day 2, and their cochleas were carefully removed by dissection. The basal, middle, and apical turns of the cochlea were used for further studies. Cochlear explants were treated with DMEM containing 10% FBS, SNAP, and EGCG (Sigma), or a combination of these, and incubated for 24 h at 33°C. The culture was then prepared for histological analysis. Organ of Corti explants were fixed for 15 min in 4% paraformaldehyde in phosphate-buffered saline (PBS). The specimens were rinsed in PBS, incubated in 0.25% Triton X-100 for 2 min, and immersed in tetramethylrhodamine isothiocyanate (TRITC)-labeled phalloidin (Sigma; 1∶100 diluted) in PBS for 20 min. After rinsing with PBS, the specimens were examined by fluorescence microscopy with the appropriate filters for TRITC (excitation: 510–550 nm; emission: 590 nm).

**Figure 8:**
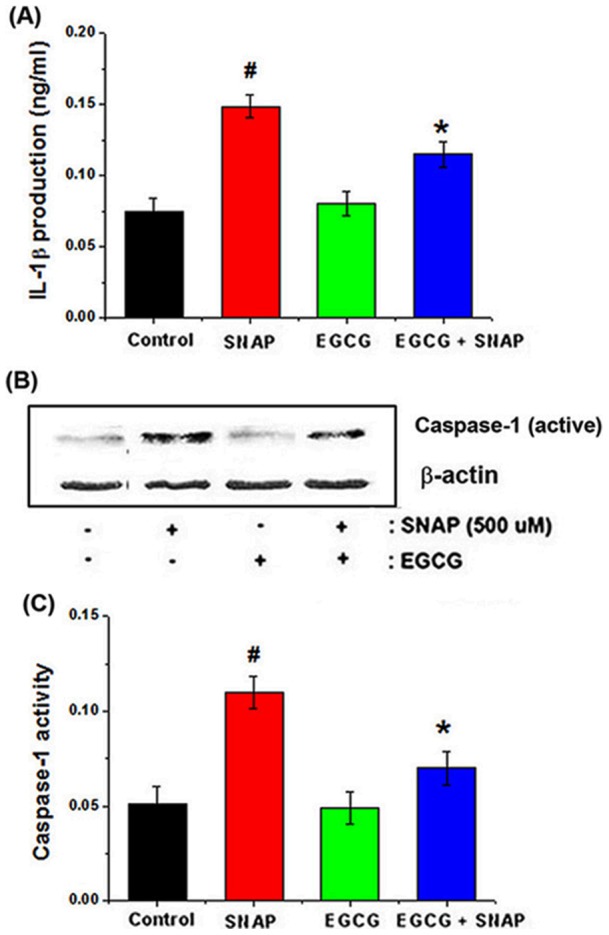
Effects of EGCG on NO-induced caspase-1 activation *in vitro* and *ex vivo.* Cells were pretreated with 50 μM EGCG, followed by treatment with 500 μM SNAP for 24 h. (A) IL-1β concentrations were measured in cell supernatants using the ELISA method. (B) Protein extracts were assayed for cleaved caspase-1 by western blot analysis. (C) Rat organ of Corti explants were pretreated with 50 μM EGCG for 2 h, followed by treatment with 500 μM SNAP. After the explants were homogenized, caspase-1 levels were confirmed using a caspase-1 assay kit. All data represent the mean ± SEM of 3 independent experiments (*^#^P*<0.05 vs. control, *P<0.05 vs. SNAP alone).

### MTT assay

To investigate the effects of NO on cell viability, SNAP was used as an NO donor. Cell viability was determined using the MTT assay as previously described [34]. Briefly, the cells (3×10^5^ cells/well) were exposed to various concentrations of SNAP (250–500 μM) or treated with SNAP at a constant concentration (500 μM) for varying periods (4–24 h). MTT solution (5 mg/mL in PBS) was added (50 μL/well), and the plates were further incubated for 4 h at 33°C. Precipitated formazan crystals were dissolved by adding DMSO. Absorption was measured using a spectrometer (Molecular Devices, Sunnyvale, CA, USA) at 540 nm.

### Flow cytometric analysis of MMP

MMP was measured using the fluorescent probe 3,3-dihexyloxacarbocyanine iodide (DiOC_6_; Invitrogen, Carlsbad, CA, USA). DiOC_6_ uptake by mitochondria is directly proportional to membrane potential. Staining intensity decreases when the reagents disrupt the MMP; quantification is based on the depolarized mitochondrial membranes. Briefly, the cells (1×10^6^ cells/dish) were cultured in the presence or absence of SNAP (250–500 μM). After trypsinization, cells were washed in PBS and incubated in DiOC_6_ (50 nM) for 30 min. The fluorescence excitation wavelength was 450–490 nm, and emission was monitored at 515–565 nm. Fluorescence intensities were analyzed by recording the Fl-1 fluorescence by flow cytometry (FACSCalibur; Becton Dickinson). Data were collected using a FACscan fluorescence-activated cell scanner with the data acquisition program QCELL Quest.

### Spectrofluorimetric measurement of intracellular ROS generation

Intracellular ROS levels were measured using the fluorescent dye 2′,7′-dichlorofluorescein diacetate (DCFH-DA). In the presence of an oxidant, DCFH is converted to a highly fluorescent molecule, 2′,7′-dichlorofluorescein (DCF). Cells were incubated with 500 μM SNAP for varying times, and then incubated for 30 min with 5 μM DCFH-DA. Fluorescence intensity was measured using a spectrofluorometer (Shimadzu Corporation, Japan) at excitation and emission wavelengths of 485 and 538 nm, respectively.

### Flow cytometry analysis

For measurement of intracellular ROS levels by flow cytometry analysis, the oxidation-sensitive probe DCFH-DA was used. NO levels were elucidated using the fluorescent NO probe DAF-2/DA. Briefly, cells were incubated with 10 μM DAF-2/DA for 30 min. For flow cytometry analysis, cells were detached by trypsinization, washed once in PBS, and resuspended in 800 μL PBS. Flow cytometric analyses (10,000 events per sample) were performed in a FACSCalibur system (BD Biosciences) with excitation and emission wavelength at 485 and 538 nm, respectively, and results were evaluated with CellQuest software.

### Assay of caspase-3 and caspse-1 activity

Enzymatic activities of caspase-3 and caspase-1 were assayed using a caspase colorimetric assay kit (R&D Systems) according to the manufacturer's protocol. Briefly, the cells were pretreated with 50 μM EGCG, treated with 500 μM SNAP for 24 h, and then lysed. The lysed cells were centrifuged at 14,000 rpm for 5 min. Protein-containing supernatants were incubated with 50 μL reaction buffer and 5 μL caspase substrates (caspase-1 or caspase-3) at 37°C for 2 h. Absorbance was measured using a plate reader at a wavelength of 405 nm. Protein was quantified using a bicinchoninic acid protein quantification kit (Sigma).

### Nitrite accumulation

Since NO itself is unstable, NO production was determined by the measurement of nitrite, a stable oxidation product of NO. Nitrite released into the media at various time points was measured by spectrophotometric assay based on the Griess reaction [35].

### Preparation of nuclear and cytoplasmic extracts

Nuclear and cytoplasmic extracts were prepared as described previously [36]. Briefly, after cell activation, cells were washed with ice-cold PBS and resuspended in 60 μL buffer A (10 mM 4-(2-hydroxyethyl)-1-piperazineethanesulfonic acid [HEPES]/KOH, 2 mM MgCl_2_, 0.1 mM ethylenediaminetetraacetic acid [EDTA], 10 mM KCl, 1 mM dithiothreitol [DTT], and 0.5 mM phenylmethylsulfonyl fluoride [PMSF], pH 7.9). The cells were allowed to swell on ice for 15 min, lysed gently with 2.5 μL of 10% Nonidet P-40, and centrifuged at 2000× *g* for 10 min at 4°C. Supernatants were collected and used as cytoplasmic extracts. Pellets containing the nuclei were resuspended in 40 μL buffer B (50 mM HEPES/KOH, 50 mM KCl, 300 mM NaCl, 0.1 mM EDTA, 10% glycerol, 1 mM DTT, and 0.5 mM PMSF, pH 7.9), left on ice for 20 min, and inverted. Nuclear debris was centrifuged at 15,000× *g* for 15 min. Supernatants (nuclear extracts) were collected, frozen in liquid nitrogen, and stored at −70°C until analysis.

### Western blot analysis

To analyze caspase-3, IκB-α, cyt *c*, caspase-1, Bcl-2, and NF-κB levels, western blot analysis was performed. The cells were rinsed with ice-cold PBS and lysed with lysis buffer (1% Triton, 1% Nonidet P-40, 0.1% sodium dodecyl sulfate [SDS], and 1% deoxycholate in PBS). Supernatants were mixed with an equal volume of 2× SDS sample buffer, boiled for 5 min, and separated on 10% SDS-polyacrylamide gels. After electrophoresis, the proteins were transferred to nylon membranes by electrophoretic transfer. Membranes were blocked for 2 h in 5% skim milk, rinsed, incubated overnight at 4°C with primary antibodies, and washed in PBS/0.5% Tween 20 (PBST) to remove excess primary Abs. Membranes were then incubated for 1 h with horseradish peroxidase-conjugated secondary Abs (anti-mouse, anti-goat, or anti-rabbit). After 3 washes in PBST, protein bands were visualized using an enhanced chemiluminescence assay (Amersham, Piscataway, NJ, USA) according to the manufacturer's instructions.

### Cytokine assay

IL-1β secretion was measured using a modification of the enzyme-linked immunosorbent assay (ELISA) described previously [37]. Ninety-six-well plates were coated with 100-μL aliquots of anti-mouse IL-1β monoclonal Abs at 1.0 μg/mL in PBS (pH 7.4) and incubated overnight at 4°C. The plates were then subjected to additional washes, and 100 μL of the cell medium or IL-1β standard was added and incubated at 37°C for 2 h. The wells were washed, followed by addition of 0.2 μg/mL of biotinylated anti-mouse IL-1β at 37°C for 2 h. After washing the wells, avidin-peroxidase was added, and plates were incubated for 30 min at 37°C. The wells were washed again, and ABTS substrate was added. Color development was measured at 405 nm by using an automated microplate ELISA reader. A standard curve was generated for each assay plate using a serial dilution of rm IL-1β.

### Transient transfection and luciferase assay

NF-κB luciferase reporter gene constructs (pNF-kB-LUC, plasmid containing NF-κB binding site; STANTAGEN, Grand Island, NY, USA) were transfected into HEI-OC1 cells using transfection reagent Tfx-50 (Promega, Madison, WI, USA) according to the manufacturer's protocol. After 24 h, the culture medium was replaced, and the cells were stimulated with SNAP. Cells were harvested after a 4-h stimulation and washed in cold PBS. After lysis, luciferase activity was measured using a luciferase assay system (Promega), normalized against β-galactosidase activity, and expressed as fold induction relative to the control. All experiments were performed in triplicate and repeated 3 times.

### Transfection with small interfering RNA

Predesigned small interfering RNAs (siRNAs) targeting NF-κB (p65) and a nonspecific control were purchased from Santa Cruz Biotechnology. Briefly, cells were grown in 6-well plates and transiently transfected with 2 μg of NF-κB or control siRNA constructs mixed with X-tremeGENE siRNA transfection reagent (Roche Applied Science, Penzberg, Germany). After incubation at 33°C and 5% CO_2_ for 24 h, cells were treated with SNAP. Gene silencing was confirmed by western blot analysis.

### NF-κB immunofluorescence

Cells were fixed with 4% paraformaldehyde and incubated with 5% bovine serum albumin (BSA) in PBS for 60 min. The preparation was incubated for 1 h at room temperature with NF-κB Abs diluted in 0.1% BSA (1∶500). Next, the preparation was washed 3 times with PBS and exposed to secondary Abs (fluorescein isothiocyanate-conjugated anti-rabbit IgG at 1∶200 and 0.1% BSA/PBS) for 60 min. For 4′,6-diamidino-2-phenylindole (DAPI) staining, cells were fixed and stained with 1 μg/mL DAPI, a DNA-specific fluorochrome, for 30 min in the dark. The fluorescent image was viewed using an Olympus confocal microscope (New Hyde Park, NY, USA).

### Data analysis

Results were expressed as the mean ± SEM of 3 independent experiments, and statistical analyses were performed by one-way analysis of variance with Tukey and Duncan *post hoc* tests to express differences between groups. All statistical analyses were performed using SPSS statistical analysis software. A *P-*value of less than 0.05 was considered statistically significant.

## Results

### Protective effects of EGCG against NO-induced destruction of hair cell arrangement in organ of Corti explants

To investigate the effect of NO on the arrangement of hair cells, organs of Corti isolated from rat cochlea at postnatal day 2 were treated with an NO donor (SNAP). In this study, SNAP was chosen as the NO donor because it is a pure NO releaser [38], [39], as opposed to SNP, which also releases toxic ONOO^−^ or cyanide ions. As shown in Fig. 1A, SNAP destroyed the orderly arrangement of the 3 outer hair cell (OHC) rows and the inner hair cell (IHC) row in the basal, middle, and apical turns. Relative cell viability is shown in Fig. 1B. SNP also affected the orderly arrangement of these hair cell rows (Fig. 1C). As shown in Fig. 1D, L-NAME (an inducible nitric oxide synthase [iNOS] inhibitor) inhibited cisplatin-induced destruction of the hair cell arrangement. In addition, we used the NO scavenger C-PTIO to confirm that NO mediated the effects of SNAP. As shown in Fig. 1E, C-PTIO inhibited SNAP-induced destruction of the hair cell arrangement. The ability of EGCG to prevent SNAP damage to the organ of Corti was also investigated. As shown in Fig. 1F, EGCG prevented SNAP-driven destruction of the arrangement of the 3 OHC rows and the IHC row. Fig. 1G shows relative hair cell viability.

### Protective effects of EGCG against NO-induced cell death in HEI-OC1 cells

To investigate the effects of SNAP on cell viability and NO levels *in vitro*, HEI-OC1 auditory cells were treated with 500 µM SNAP for varying times. SNAP affected cell viability in both time- and dose-dependent manners (Fig. 2A and B). As shown in Fig. 2A, low doses of SNAP (<100 μM) did not affect cell viability. SNAP exposure also resulted in time-dependent cell damage along with a marked increased in NO production (Fig. 2C). Moreover, we found that EGCG exerted a significant protective influence against SNAP-induced cell death (Fig. 2D), thereby providing an insight into the mechanism mediating NO-induced ototoxicity in auditory cells treated with SNAP for 24 h.

### Protective effects of EGCG on NO-induced MMP loss in HEI-CO1 cells

To determine the effects of SNAP on mitochondrial membrane integrity, cells were incubated with SNAP (250–500 μM) for 24 h, after which MMP levels were measured as an index of mitochondrial membrane integrity. The cells were loaded with DiOC_6_, an MMP-dependent fluorescent probe, and the resulting fluorescence was measured by flow cytometry. Compared to the control, DiOC_6_ fluorescence intensity decreased following SNAP exposure (left-shifting of the cell distribution; Fig. 3A). Fig. 3B shows relative fluorescence levels as a function of SNAP concentration. Furthermore, we demonstrated that EGCG inhibited SNAP-induced MMP loss (Fig. 3C). Relative fluorescence levels are presented in Fig. 3D.

### Protective effects of EGCG on NO-induced ROS generation in HEI-CO1 cells

To investigate the effects of SNAP on intracellular ROS generation, HEI-OC1 auditory cells were treated with 500 µM SNAP for various times. ROS production increased after SNAP exposure, but the effect became less pronounced as exposure time increased (Fig. 4A). EGCG effectively suppressed the SNAP-induced increase in ROS levels (Fig. 4B). We confirmed the effects of EGCG on ROS levels by using flow cytometry (Fig. 4C). Additionally, we used DAF-2/DA fluorescence to measure NO levels. As shown in Fig. 4D, EGCG significantly attenuated the SNAP-induced increase in NO levels in HEI-OC1 auditory cells.

### Regulatory effects of EGCG on NO-induced apoptosis-related gene expression in HEI-CO1 cells

Western blot analysis was performed to assess the effects of SNAP on the release of cyt *c* into the cytosol. SNAP induced the release of cyt *c* into the cytosol, and EGCG inhibited this process (Fig. 5A). The relative quantity of cyt *c* was determined using an image analyzer (Fig. 5B). As shown in Fig. 5C, EGCG also inhibited the reduction in Bcl-2 levels induced by SNAP. Relative Bcl-2 expression is shown in Fig. 5D. Next, we performed western blotting and a caspase-3 activity assay to determine whether NO-induced apoptosis was associated with the regulation of caspase-3 activity. SNAP increased the expression of caspase-3 (active form), while EGCG effectively inhibited this increase (Fig. 5E). EGCG also attenuated the SNAP-induced increase in caspase-3 activity (Fig. 5F).

### Protective effects of EGCG on NO-induced NF-κB signaling in HEI-CO1 cells

To determine the association of NO-induced apoptosis with the NF-κB pathway, we silenced endogenous NF-κB using specific siRNA. The siRNA effectively inhibited NF-κB expression in the nucleus relative to control cultures transfected with scrambled siRNA (Fig. 6A). As shown in Fig. 6B, knockdown of NF-κB was effective at inhibiting SNAP-induced caspase-3 activation (as an apoptosis marker). The siRNA transfections resulted in 52% and 48% knockdown of NF-κB and caspase-3, respectively (Fig. 6C). Based on these findings, we investigated the relationship between the protective mechanisms of EGCG and regulation of the NF-κB pathway. Our results revealed that SNAP induced the degradation of IκB-α in the cytosol and translocation of NF-κB into the nucleus; EGCG suppressed these SNAP-induced phenomena (Fig. 6D). Next, we performed a luciferase assay to investigate the effects of EGCG on NF-κB promoter activity. As shown in Fig. 6E, SNAP treatment enhanced NF-κB promoter activity, while EGCG pretreatment inhibited this SNAP-induced increase in NF-κB promoter activity. Immunofluorescent staining of NF-κB (green) and nuclei (blue) revealed that SNAP treatment caused translocation of NF-κB into the nucleus, while pretreatment with EGCG inhibited this phenomenon (Fig. 6F).

### Protective effects of EGCG on NO-induced NF-κB activation in organ of Corti explants

Next, we investigated the regulatory effects of SNAP on NF-κB activation *ex vivo*. As shown in Fig. 7, treatment with SNAP induced NF-κB activation in the organ of Corti, and EGCG inhibited SNAP-induced NF-κB activation (red).

### Protective effect of EGCG on NO-induced caspase-1 activation in HEI-CO1 cells and organ of Corti explants

We investigated whether NO-mediated ototoxicity occurred via the production of IL-1β and activation of caspase-1. As shown in Fig. 8A and B, SNAP induced IL-1β production and increased the levels of caspase-1 (cleaved form) in HEI-CO1 cells, while EGCG inhibited these effects. To confirm the effects of EGCG on caspase-1 activation *ex vivo*, we performed a caspase-1 activity assay in organ of Corti explants. The results demonstrated that SNAP induced caspase-1 activation, and this effect was again inhibited by EGCG (Fig. 8C).

## Discussion

We have shown, for the first time, that EGCG is effective in preventing the destruction of hair cell arrays and apoptosis both *in vitro* and *ex vivo*. EGCG is also effective in counteracting ototoxicity by suppressing NF-κB and caspase-1 activation.

EGCG is the main constituent of polyphenols and the most abundant and active polyphenolic compound with potent biological properties, including antioxidant, hepatoprotective, chemopreventive, and anticarcinogenic effects. It has been reported that the active site of EGCG can react with oxygen free radicals, supporting that EGCG possesses potent antioxidant properties [40]. In addition, EGCG is a known inhibitor of the STAT1 transcription factor, which has been implicated in the production of ROS and the activation of caspase-3 in cisplatin-induced ototoxicity [41]. However, the effects of EGCG on NO-induced ototoxicity have not yet been established.

NO is a free radical that predominantly functions as a messenger and effector molecule. Many studies have suggested that free oxygen radicals can cause hearing impairment. Recent evidence suggests that excessive NO production plays an important role in pathological damage of the cochlea and elevated hearing thresholds [42]. The induction of apoptotic cell death by NO depends on its concentration and the cell type involved. High concentrations of NO donors have been shown to generate toxic concentrations of NO and induce apoptosis. However, NO treatment at lower, more physiological levels may often have protective effect, preventing the onset of apoptosis in many mammalian cells [43], [44]. In the current study, we found that higher concentrations of SNAP (>250 mM) induced auditory cell death, but low doses of SNAP (<100 µM) did not affect cell viability. This finding is consistent with other studies that have demonstrated the induction of Molt-4 cell death by high concentrations of SNAP [45]. Many studies have shown that the ototoxicity of cisplatin can be mediated by increased NO production in the inner ear, leading to auditory cell destruction. L-NAME, a competitive inhibitor of NOS, was shown to reduce cisplatin-induced hearing disturbances [46]. In this study, we confirmed that L-NAME suppressed cisplatin-induced hair cell destruction and iNOS expression in organ of Corti explants (data not shown), and we investigated the direct effects of NO and protective effects of EGCG on hair cell death. NO destroyed the orderly arrangement of the 3 OHC rows and the IHC row in the basal, middle, and apical turns in Corti explants, and EGCG abrogated NO-induced destruction of hair cell arrays. Additionally, an NO scavenger effectively inhibited NO-induced hair cell destruction. These results imply that a high concentration of NO is involved in ototoxicity and that this phenomenon can be counteracted by antioxidants.

In mammals, mitochondria act as the central checkpoint for many forms of apoptosis. The mitochondrial pathway is believed to be the main target for survival signaling pathways [47]. NO has been reported to interfere with the mitochondrial respiratory chain at several sites, resulting in increased generation of ROS that subsequently react with NO to form peroxynitrite, which in turn damages cells and leads to cell death. Mitochondrial alterations leading to mitochondrial membrane depolarization induce apoptosis by reduction of MMP and release of cyt *c*. Thus, we investigated NO-induced cell death, MMP loss, ROS generation, and cyt *c* release in auditory HEI-OC1 cells. The results revealed that NO-induced ROS production may lead to a decrease in MMP, which in turn increases mitochondrial membrane permeability and releases mitochondrial apoptogenic factors, such as cyt *c*, into the cytosol. This indicated that NO-induced apoptosis may occur through the mitochondrial pathway. Moreover, EGCG regulated the NO-mediated mitochondrial pathway in auditory cells. These findings demonstrate that the antiapoptotic effects of EGCG on NO-induced apoptosis may be related to its antioxidant potential and its ability to scavenge ROS. However, the mechanisms through which NO triggers other pathways in auditory cells were not examined in this study. Therefore, further studies are needed to identify nonmitochondrial signaling pathways in NO-induced ototoxicity.

Many recent studies have investigated the association between NF-κB activation and hearing loss. Some have suggested that NF-κB family proteins found in the inner ear are required for normal hair cell function [49], while others have reported that signal transduction pathways respond rapidly to ototoxic stimulants, such as noise exposure and ototoxic drugs [19], [20]. The activation of NF-κB induces cochlear lateral wall insults by producing large amounts of ROS [21], [23]. Acoustic overstimulation also increases the expression of inflammatory factors through NF-κB activation in the inner ear [24]. Despite the results of these studies, the functional role of NF-κB in hearing loss remains controversial. Moreover, the ability of NO to regulate NF-κB can vary with cell type, NO concentration, and duration of exposure. Some studies have suggested that SNP induces NF-κB activation, as was demonstrated by cytosolic IκB-α phosphorylation and degradation in human periodontal ligament cells [50]. Others have reported that NO-induced apoptosis is a result of downregulation of NF-κB DNA-binding activity, as shown in J774 macrophages [51]. In this study, we sought to determine whether the cytotoxic effects of NO were exerted through the regulation of the NF-κB pathway. The results showed that NO induced the degradation of IκB-α in the cytosol and translocation of NF-κB to the nucleus in HEI-OC1 cells. To test this phenomenon *ex vivo*, we used rat organ of Corti explants to confirm that NO caused NF-κB activation. Silencing NF-κB with specific siRNA inhibited NO-induced apoptosis, and pretreatment with EGCG suppressed the degradation of IκB-α and translocation of NF-κB to the nucleus. These results suggested that the cytotoxicity of NO was mediated by NF-κB activation both *in vitro* and *ex vivo*. Accumulating evidence has shown that the association of NF-κB activation with apoptosis-related gene expression depends on cell type. Moreover, Bcl-2 proteins control the release of mitochondrial cyt *c* by regulating mitochondrial permeability. Recent studies have shown that NF-κB acts upstream of apoptosis-related genes, including Bcl-2 [52]. In this study, we found that treatment with an NO donor inhibited Bcl-2 expression. Bcl-2 is a marker for antiapoptotic activity and a product of one of the NF-κB target genes. Thus, we postulated that NF-κB may regulate apoptosis-related genes in NO-mediated cytotoxicity.

Caspases serve important functions in apoptosis and have been implicated in NO-induced cell death [48]. In this study, we demonstrated that NO enhanced caspase-3 activity, while EGCG attenuated caspase-3 activation in auditory cells. Therefore, the mechanism mediating NO-induced apoptosis in auditory cells may, at least in part, involve a caspase-dependent pathway. Although NO can induce apoptosis through a caspase-dependent pathway, the effects of NO on caspase-independent processes were not elucidated in the present study. Hence, further studies are needed to determine how NO influences translocation of AIF from the cytosol to the nucleus and how NO mediates caspase-independent apoptosis. Caspase-1 is an IL-1-converting enzyme involved in numerous biological processes, including apoptosis and inflammation. Work by Zhang et al. has indicated that caspase-1 triggers the release of cyt *c* and activation of caspase-3 in ischemia/hypoxia-mediated neuronal cell death [53]. Studies have also shown that cisplatin induces the activation of caspase-1 in cochlear hair cells and spiral ganglion neurons [28]. In this study, we found that NO treatment resulted in caspase-1 activation and IL-1β production, while EGCG inhibited the observed NO-induced increase in IL-1β production and caspase-1 activation, suggesting that the caspase-1 pathway is a potential therapeutic target for preventing NO-induced ototoxic damage. Receptor interacting protein (RIP)-2, specific adaptor, has been found to regulate the activation of caspase-1; the caspase activation and recruitment domains (CARDs) of RIP-2 bind to the CARD of the caspase-1 prodomain via CARD–CARD interactions, inducing caspase-1 activation. This RIP-2/caspase-1 interaction causes IKK phosphorylation and IκB-α degradation. Thus, NF-κB is released and translocates to the nucleus, where it induces gene transcription [54]. Caspase-1 may also contribute to NF-κB activation through the autocrine action of IL-1β [55]. From this, we postulated that the NF-κB pathway may be involved in caspase-1 activation in auditory cells. However, further studies will be needed to clarify the precise relationship between NF-κB and caspase-1 in NO-mediated ototoxicity. Furthermore, we demonstrated that the antiapoptotic mechanism of EGCG may be driven by the regulation of the signaling molecules that participate in the NO-mediated apoptotic process.

In conclusion, high levels of NO resulted in cell death, ROS generation, MMP loss, cyt *c* release, and caspase-3 activation in auditory cells. In addition, NO destroyed hair cells in the basal, middle, and apical cochlear turns in primary organ of Corti explants from rats. NO ototoxicity was mediated through the activation of NF-κB and caspase-1, and EGCG was effective in counteracting this ototoxicity by suppressing NF-κB and caspase-3 activation and preventing hair cell array destruction. This study therefore indicates that EGCG may be a beneficial agent for preventing or halting the progression of certain types of hearing loss.
